# Role of light in eye growth and light as a therapy for myopia control

**DOI:** 10.3389/fopht.2026.1812006

**Published:** 2026-08-05

**Authors:** Neeraj K. Singh, Jesson Martin

**Affiliations:** Kentucky College of Optometry, University of Pikeville, Pikeville, KY, United States

**Keywords:** chromatic aberration, emmetropization, light therapy, myopia, red light

## Abstract

The escalating global prevalence of myopia—projected to affect nearly 5 billion individuals by 2050—represents one of the most pressing public health challenges of the 21st century. This comprehensive review synthesizes current evidence on light-mediated mechanisms governing ocular growth and their therapeutic explorations for myopia prevention and control. We examine how specific wavelengths, illuminance levels, and temporal exposure patterns influence emmetropization through chromatic aberration processing, photobiomodulation, and circadian regulation. This review provides a mechanistic framework for understanding how environmental light exposures shape refractive development and offers evidence-based guidance for implementing light-based interventions in clinical practice. As myopia transitions from a simple refractive error to a complex, multifactorial disease, these biologically informed approaches represent a paradigm shift toward prevention-focused management strategies.

## Introduction

Myopia has evolved from a relatively benign refractive condition to a sight-threatening disease of pandemic proportions. Global prevalence has surged dramatically from 22.9% in 2000 to approximately 36% in 2023, with projections indicating that 39.8% of children and adolescents worldwide—equivalent to nearly 740 million individuals—will be myopic by 2050 (1). The burden extends beyond simple refractive error, as high myopia (≤-6.00 D) predisposes to irreversible pathological complications including myopic maculopathy, retinal detachment, and glaucoma (2). The anatomical basis of myopia involves excessive axial elongation relative to the eye’s optical power, typically resulting from dysregulated scleral remodeling during critical developmental periods (3). While genetic predisposition contributes substantially to myopia risk, the temporal and geographic variations in prevalence strongly implicate environmental factors, particularly those related to visual experience and light exposure (4–6).

Emmetropization—the process by which the developing eye achieves precise optical focus—operates through various feedback mechanisms that compare retinal image quality with optimal visual requirements (6). This system demonstrates extraordinary precision, typically achieving refractive accuracy within ±0.50 D despite enormous individual variations in ocular component dimensions (6). However, mounting evidence suggests that modern environmental conditions may overwhelm these regulatory mechanisms, leading to the myopia epidemic observed in urbanized populations (5, 7–9).

Recent advances in understanding the mechanisms underlying light-mediated eye growth control have revealed previously unrecognized therapeutic targets. The discovery that retinal processing of chromatic aberration guides emmetropization has revolutionized our conceptual framework for myopia development (10). Similarly, identification of specific photoreceptor pathways mediating wavelength-dependent growth responses has enabled development of targeted interventions demonstrating some clinical efficacy (11).

This review synthesizes current knowledge regarding light-mediated regulation of ocular growth, with particular emphasis on translational applications for myopia prevention and control. We examine the optical mechanisms through which different spectral compositions, intensities, and temporal patterns of light exposure influence refractive development, and evaluate emerging therapeutic strategies. This review was conducted as a narrative synthesis of the published literature. A systematic search was performed across PubMed/MEDLINE, Scopus, and Web of Science using the following primary search terms and their combinations: “myopia, “ “ocular growth, “ “light therapy, “ “repeated low-level red light, “ “chromatic aberration, “ “emmetropization, “ “retinal dopamine, “ “outdoor light exposure, “ “myopia control, “ and “refractive development.” The search encompassed English-language articles from January 2000 to April 2026, with inclusion of seminal earlier works where appropriate. Articles were screened by title and abstract, with full-text review for potentially eligible studies, prioritizing RCTs, systematic reviews, meta-analyses, and mechanistic studies. Given the narrative design, formal PRISMA reporting was not applied.

## Optical foundations & chromatic aberration

**Figure 1:**
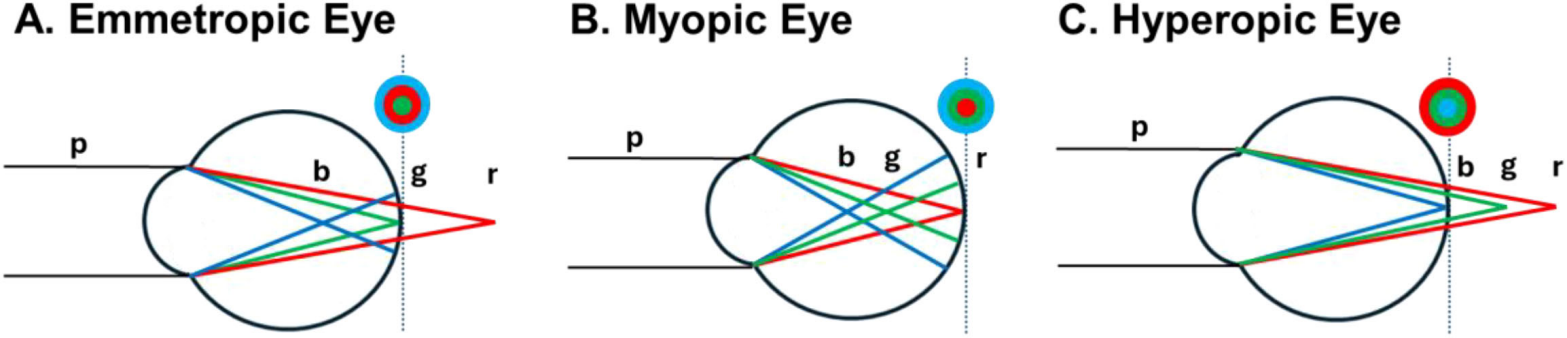
Schematic of longitudinal chromatic aberration (LCA). Panels illustrate differential focal positions of blue, green, and red wavelengths under emmetropic **(A)**, myopic **(B)**, and hyperopic **(C)** conditions.

The human eye functions as an intricate optical system in which refractive power is distributed across multiple media—the cornea, crystalline lens, aqueous humor, and vitreous body. Each medium contributes a wavelength-dependent refractive index, yielding longitudinal chromatic aberration (LCA), whereby shorter (blue) wavelengths focus anterior to longer (red) wavelengths (**Figure 1**). Early *in vivo* measures indicate that LCA spans approximately 1.62 diopters (D) between 472 nm and 652 nm in infant eyes, compared with 0.96 D in adults, reflecting developmental changes in ocular power and axial length (12). This 1.7-fold elevation in infant LCA arises from the combination of higher corneal curvature and lens power during early postnatal growth, yielding more pronounced chromatic focal shifts that constitute robust directional cues for emmetropization (13).

**Figure 1** illustrates the concept and effects of longitudinal chromatic aberration (LCA) in the human eye. Panel A depicts the principle of LCA, where polychromatic light entering the eye is dispersed into its component wavelengths. This dispersion results in different focal points for different wavelengths, with shorter wavelengths (blue) focusing in front of the retina and longer wavelengths (red) focusing behind it. The middle wavelengths (green) ideally focus on the retina. Panels B and C illustrate the impact of LCA under conditions of myopic and hyperopic defocus, respectively. Panel B shows an overpowered (myopic) eye where the red wavelengths are in best focus on the retina, while the blue and green wavelengths are focused in front of the retina. Panel C depicts an underpowered (hyperopic) eye where the blue wavelengths are in best focus on the retina, with the green and red wavelengths focused behind it.

Quantifying longitudinal chromatic aberration (LCA) in human eyes relies on both psychophysical and objective techniques. Psychophysical methods employ monochromatic stimulus modulation to derive spectral defocus curves, while objective approaches use wavefront sensors or double-pass retinal imaging to map point-spread functions across wavelengths (12, 14–17). These measurements reveal that the eye’s LCA closely matches theoretical predictions based on refractive indices of constituent ocular media (18), though individual variability arises from differences in lens gradient refractive index and age-related changes in lens shape.

Beyond LCA, in addition to the higher order monochromatic aberrations the eye also exhibits transverse chromatic aberration (TCA) (**Table 1**), in which off-axis rays of varying wavelengths refract differently, producing laterally shifted chromatic fringes in the peripheral visual field (19). TCA magnitude increases with field angle, reaching several arcminutes at peripheral eccentricities of 20–30 degrees (19, 20). Recent investigations demonstrate that TCA can alter local image quality and may influence peripheral emmetropization signals (21). Computational modeling suggests that the combination of TCA and higher-order monochromatic aberrations creates “chromatic optical anisotropy, “ unique wavelength-dependent blur patterns whose orientation informs the sign of defocus in off-axis retina (22, 23).

**Table 1:** Key optical aberrations and measurement methods.

Aberration type	Characteristics	Magnitude (central)	Measurement techniques	Functional role
Longitudinal Chromatic Aberration (LCA)	Wavelength-dependent axial focal shift.	Approximately 1.62 D in infants and 0.96 D in adults between 472 and 652 nm	Monochromatic defocus curves; wavefront sensing	Provides directional cues for accommodation and growth
Transverse Chromatic Aberration (TCA)	Wavelength-dependent lateral image shift.	~1–3 arcmin at 20-30	Double-pass imaging; adaptive optics	Influences peripheral image quality and defocus detection
Higher-order Monochromatic Aberrations (HOAs)	Non-paraxial refractive errors (coma, spherical)	Varies by individual	Shack-Hartmann aberrometry; ray tracing	Affects overall retinal image contrast and signaling

**Figure 2:**
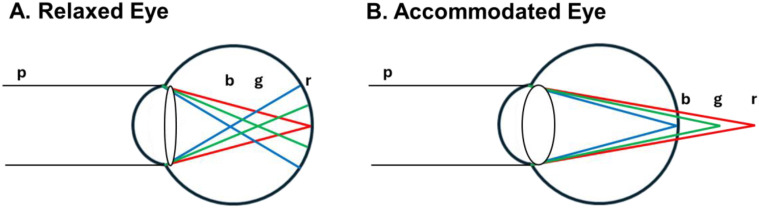
Schematic of LCA on the accommodative mechanism. Panel **(A)** shows how polychromatic light disperses into different wavelengths, with long wavelength red focusing on the retina, mid and short wavelengths focusing in front of the retina in a relaxed eye. Panel **(B)** shows an accommodated eye with blue wavelengths on the retina and green/red wavelengths behind.

The optical design of the eye integrates LCA with accommodation mechanisms (24). Accommodation involves zonular fiber modulation of lens curvature to shift focal planes (**Figure 2**), yet chromatic cues provide directionality: blue-dominant blur indicates hyperopic defocus (accommodation insufficient), while red-dominant blur indicates myopic defocus (accommodation excessive) (13). Studies report that retinal neurons encoding contrast spectra modulate choroidal and scleral growth signals, linking optical aberration patterns to physiological growth adjustments (24–26). Chromatic aberration’s role extends to myopia control strategies.

Next-generation myopia control spectacle lenses (27) and multifocal contact lenses (28–30) aim to slow myopia progression by introducing simultaneous peripheral myopic defocus through discrete optical power zones, while preserving central image quality. This optical strategy is thought to provide retinal cues that inhibit axial elongation, independent of chromatic or spectral manipulation (31). Clinical trials with these myopia control lenses show approximately 50% reduction in myopia progression in children, underscoring the translational potential of defocus manipulation (32). The optical properties of these myopia control lenses—including their chromatic transmission characteristics and the differential focusing of short and long wavelengths across central and peripheral zones—are directly governed by the LCA framework described above. Understanding how these lenses interact with wavelength-dependent defocus is therefore essential for appreciating their mechanism of action.

## Retinal processing mechanisms

Retinal circuitry translates optical aberrations into biochemical and biophysical signals that govern axial growth. This section details the photoreceptor and inner-retinal pathways—cone subtypes, intrinsically photosensitive retinal ganglion cells (ipRGCs), and non-visual opsins—that encode spectral defocus, brightness, and temporal patterns to modulate emmetropization.

### Cone photoreceptor contributions and chromatic signal integration

Human cones segregate into short- (S), medium- (M), and long-wavelength (L) sensitive types, each tuned to ~420 nm, 530 nm, and 560 nm peaks respectively (33). Spatial and temporal integration of their outputs enables detection of chromatic aberration. Psychophysical studies demonstrate that selective S-cone contrast drives changes in accommodation and choroidal thickness consistent with myopic versus hyperopic defocus (31). Electrophysiological recordings in macaque retina reveal S-cone–driven pathways projecting through koniocellular layers of the lateral geniculate nucleus, linking chromatic contrast to higher-order growth regulation centers (10). Dopaminergic amacrine cells receive convergent input from L- and M-cone circuits and modulate gap junction coupling to adjust signal integration under varying spectral conditions. In chicks, pharmacological blockade of S-cone circuitry abolishes chromatic aberration–induced growth responses, while selective enhancement of S-cone contrast restores normal emmetropization, confirming the indispensable role of S-cone signals (34).

### Intrinsically photosensitive retinal ganglion cells and brightness coding

ipRGCs express melanopsin and respond to blue-light spectral irradiance (~480 nm) independently of rod/cone input. They convey irradiance and circadian information to suprachiasmatic and olivary pretectal nuclei and modulate retinal dopamine synthesis (35). In mice, melanopsin knockout reduces bright-light–mediated suppression of form-deprivation myopia, implicating ipRGC-driven dopamine release as a crucial stop signal for axial elongation (10). Human evidence that blue-light stimulation directly increases retinal dopamine is indirect; unsupported claims that blue-light goggles acutely increase vitreous dopamine metabolites in humans should not be made (36). Blue-light and melanopsin pathways remain mechanistically plausible but require careful distinction between animal biochemical evidence and human clinical endpoints (36).

### Neuropsin (OPN5) and violet light–specific pathways

OPN5 is a UV-sensitive opsin (peak λ ≈380 nm) expressed in a subset of retinal ganglion cells distinct from ipRGCs. In mouse models, OPN5 activation by violet light upregulates early growth response-1 (EGR1) in inner retinal neurons, initiating transcriptional programs that slow axial elongation (37). OPN5 knockout abolishes violet-light protection against lens-induced myopia, demonstrating pathway specificity (38). Single-cell RNA sequencing of human retina reveals OPN5-positive RGC clusters co-expressing EGR1 and adenylate cyclase genes, providing a molecular basis for violet light–mediated cAMP signaling in growth control (39). It is important to note that OPN5-mediated violet light protection has been established primarily in murine models; whether analogous pathways operate equivalently in the human retina remains to be determined in controlled clinical studies.

### Retinal dopamine signaling cascade

**Figure 3:**
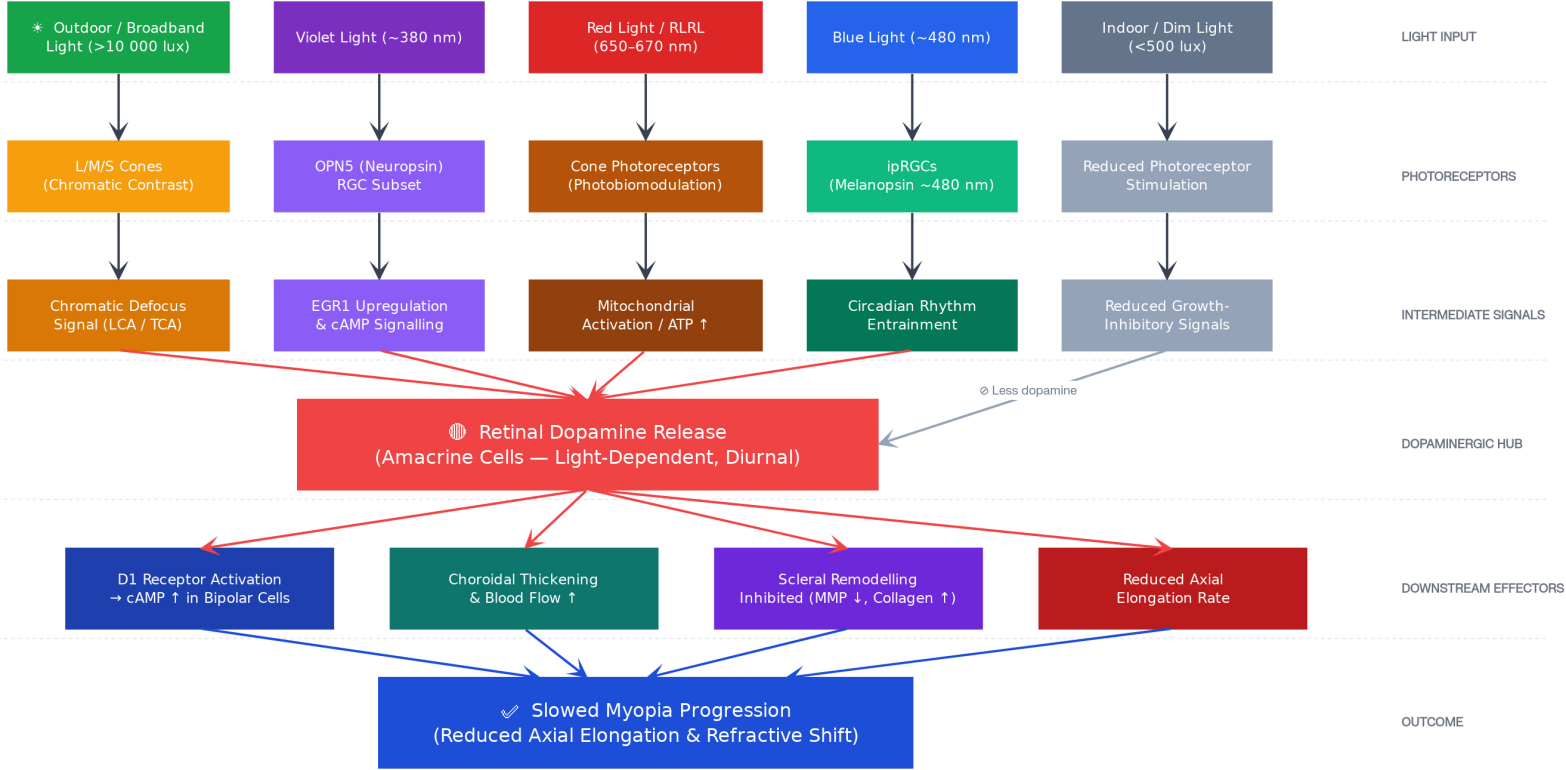
Retinal signalling cascade linking spectral light exposure to inhibition of axial elongation and myopia control. Six functional tiers are illustrated (1). Light input: outdoor broadband light (>10, 000 lux), violet (~380 nm), red/RLRL (650–670 nm), blue (~480 nm), and indoor dim light (<500 lux). (2) Photoreceptors: L/M/S cones (chromatic contrast), OPN5-expressing RGCs (violet light), cone photoreceptors (photobiomodulation), and ipRGCs (melanopsin, ~480 nm). (3) Intermediate signals: chromatic defocus (LCA/TCA), EGR1/cAMP signalling, mitochondrial ATP production, and circadian entrainment. (4) Dopaminergic hub: all active pathways converge on light-dependent retinal dopamine release by amacrine cells; dim indoor light (dashed arrow, ⊘) results in attenuated dopamine output. (5) Downstream effectors: D1 receptor–mediated cAMP elevation, choroidal thickening, and scleral remodelling inhibition (MMP↓/collagen↑). (6) Outcome: reduced axial elongation and slowed myopia progression. Dashed arrow denotes inhibitory or attenuated signalling. cAMP, cyclic adenosine monophosphate; EGR1, early growth response protein 1; ipRGCs, intrinsically photosensitive retinal ganglion cells; LCA, longitudinal chromatic aberration; MMP, matrix metalloproteinase; OPN5, neuropsin; RGC, retinal ganglion cell; RLRL, repeated low-level red light; TCA, transverse chromatic aberration.

Dopamine serves as the principal neuromodulator coupling light exposure to eye growth. Synthesized by tyrosine hydroxylase–expressing amacrine cells, dopamine release is light intensity–dependent and exhibits diurnal rhythms, peaking during daytime (40). Binding to D1 receptors on ON-pathway bipolar cells increases intracellular cAMP, inhibiting pathways that promote scleral matrix remodeling (41). Pharmacological blockade of D1 receptors prevents bright-light suppression of form-deprivation myopia in chicks, while D2 receptor modulation alters the magnitude but not the direction of growth responses (42). Dopamine antagonists also abrogate the protective effects of the Repeated Low-Level Red Light (RLRL) and outdoor light, underscoring dopamine’s central role across modalities (43). The integrated retinal signalling cascade linking light exposure to axial elongation control is summarized in **Figure 3**.

## Animal models of spectral light effects on ocular growth

Animal models have been indispensable for elucidating the mechanisms through which light spectra influence eye growth. While no single model fully replicates human emmetropization, complementary insights from chicks, tree shrews, mice, and non-human primates have clarified species-specific responses and conserved pathways.

### Chick models

Chicks exhibit rapid ocular growth and robust visual-environment responses, making them a foundational model. Chromatic simulations of myopic and hyperopic defocus show that chick eyes can compensate in appropriate directions, supporting the hypothesis that LCA provides a directional cue for ocular growth (25). Bright-light experiments in chicks also demonstrate that intermittent episodes of high illuminance can suppress experimental myopia more effectively than continuous bright light, highlighting the importance of temporal exposure patterns (45). Dopamine is repeatedly implicated in avian light responses, and ambient illuminance can alter retinal dopamine release and refractive development in chicks (40, 46, 52). Because avian retinas contain violet/UV-, blue-, green-, and red-sensitive single cones plus double cones, chick findings should be interpreted as evidence for chromatic growth regulation in a tetrachromatic retinal system rather than as direct analogues of human trichromatic processing.

### Tree shrews and non-human primates

Tree shrews, phylogenetically closer to primates, exhibit wavelength-specific growth responses similar to humans. Exposure to long-wavelength (red) light produces hyperopic shifts and prevents lens-induced myopia, whereas short-wavelength (blue/green) light lacks protective effects (47). Narrow-band cyan (500 nm) fails to maintain emmetropia despite optical focus, underscoring the necessity of full-spectrum signals for normal development (48). Rhesus monkeys demonstrate that broad long-wavelength lighting slows axial elongation and induces hyperopia. Chronic exposure to 600–700 nm light reduces form-deprivation myopia by ≈40%, confirming translational relevance for human repeated low-level red light (RLRL) therapy (49). Spectral effects are independent of illuminance when photon density is matched, highlighting wavelength composition rather than intensity as the key variable (50).

### Mouse models

Mice, despite diurnal nocturnality and limited visual acuity, provide genetic tractability for dissecting molecular pathways. Bright-light exposure (2, 500–5, 000 lux) suppresses form-deprivation myopia through D1 receptor–mediated dopamine signaling in ON-pathway bipolar cells (51). Both scotopic (<1 lux) and photopic (>1, 000 lux) lighting afford protection, whereas mesopic (1–100 lux) conditions permit myopia—revealing a U-shaped illuminance–response curve (52). Genetically modified mice lacking OPN5 lose violet-light–dependent myopia protection, and melanopsin knockouts lose bright-light responses, confirming photoreceptor-specific pathways. Single-cell RNA sequencing identifies cell-type–specific expression changes in dopamine, retinoic acid, and hypoxia pathways during spectral manipulations (53).

## Clinical trials and therapeutic applications

Translating basic insights into clinical practice has yielded a diverse therapeutic toolkit for myopia control. This section elaborates on the randomized controlled trials (RCTs), meta-analyses, and safety studies of spectral light interventions, optical defocus lenses, pharmacological agents, and combination strategies.

### Repeated low-level red light therapy

A multicenter RCT in China enrolled 264 children aged 8–13 years with cycloplegic spherical equivalent refraction (SER) from -1.00 to -5.00 D, randomizing participants to twice-daily 3-minute sessions of 650 nm RLRL plus single-vision spectacles versus single-vision spectacles alone (54). At 12 months, adjusted axial elongation was 0.13 mm (95% CI, 0.09–0.17) in the RLRL group versus 0.38 mm (95% CI, 0.34–0.42) in controls, with a between-group difference of 0.26 mm (95% CI, 0.20–0.31) (54). Adjusted 12-month SER progression was -0.20 D (95% CI, -0.29 to -0.11) with RLRL versus -0.79 D (95% CI, -0.88 to -0.69) with single-vision spectacles, corresponding to a 0.59 D between-group difference in favor of RLRL (54). A separate RCT in children and adolescents with high myopia reported reduced axial elongation over 12 months, but its clinical interpretation remains bounded by sample characteristics and follow-up duration (55). However, the existing evidence base for RLRL therapy carries important limitations that warrant caution in clinical translation. Nearly all published RCTs have been conducted in East Asian pediatric populations—primarily children in China—raising questions about generalizability to other ethnic groups, refractive error distributions, and environmental contexts. No prospective trial has yet provided follow-up data beyond five years, leaving long-term axial length control, rebound effects, and cumulative retinal safety unresolved. The controversy surrounding retinal safety—including emerging case reports of outer retinal disruption and photoreceptor density changes identified by adaptive optics imaging—underscores the need for continued post-market surveillance and independent replication in ethnically diverse cohorts before widespread clinical adoption can be recommended.

Meta-analyses consolidating seven RCTs (N, 1, 031) confirm RLRL’s superior performance over orthokeratology (42% greater axial control) and low-dose atropine (4% greater control) (56). Safety profiles across 2, 380 participants report an adverse event rate of 0.09 per 100 patient-years, predominantly transient afterimages lasting <10 minutes (57). High-resolution adaptive optics imaging revealed minor cone density reductions in <2% of subjects after 24 months, the clinical significance of which remains under investigation (58). Direct comparisons of repeated low-level red light (RLRL) therapy with atropine, orthokeratology, and defocus incorporated multiple segments (DIMS) lenses must be interpreted with caution. Meta-analytic estimates of superiority are largely derived from indirect network comparisons rather than head-to-head RCTs, and are subject to substantial heterogeneity from differences in participant age, baseline myopia severity, treatment duration (6–24 months), outcome definitions, and adherence monitoring. RLRL trials have predominantly enrolled East Asian children aged 8–13 years, while atropine and orthokeratology trials have broader geographic representation. Until adequately powered multi-arm RCTs compare these interventions directly in diverse populations over ≥3 years, meta-analytic rankings should be considered hypothesis-generating rather than definitive evidence of superiority. **Table 2** provides for a comprehensive comparison of myopia control interventions.

**Table 2:** Comparison of current myopia control interventions.

Intervention	Mechanism	Efficacy	Safety	Rebound	Regulatory status	Evidence (GRADE)
RLRL (650–670 nm)	Putative photobiomodulation; retinal/choroidal signaling; dopamine involvement remains incompletely proven in humans	Jiang 2022: adjusted axial elongation 0.13 mm (95% CI, 0.09–0.17) vs 0.38 mm (95% CI, 0.34–0.42) at 12 months	Transient afterimages common; systematic review found 0.088 AEs/100 patient-years; adaptive-optics cohort found lower paracentral cone density in some users	Unclear; no long-term cessation data beyond limited follow-up	China: Class III after July 2024 regulatory changes; U.S.: no FDA-authorized RLRL myopia device identified; Australia: Eyerising ARTG 412752 advertising representations permitted subject to TGA conditions; EU/NZ status product-specific	**Moderate** — short-term RCTs mainly in East Asian children; long-term safety uncertain
Orthokeratology	Overnight corneal reshaping; peripheral myopic defocus; possible accommodative and retinal signaling effects	Approximately 40–55% axial-elongation reduction across studies	Corneal staining; lens binding; rare microbial keratitis; hygiene dependent	Moderate; axial elongation may resume after cessation	FDA-cleared overnight corneal reshaping lenses for correction; myopia-control claims vary by jurisdiction/product	**Moderate–High** — multiple RCTs and long clinical experience, but ocular-surface risk requires monitoring
DIMS spectacle lenses	Simultaneous myopic defocus using +3.50 D segments surrounding central distance zone	52% reduction in myopia progression and 62% reduction in axial elongation	Mild adaptation blur; no serious ocular AEs in pivotal RCT	Low to moderate; less rebound concern than atropine	Not FDA-authorized (US); CE-marked (EU); approved in ≥30 markets including Hong Kong SAR, mainland China, Canada, UK, Japan, and Australia	**Moderate** — RCT evidence, predominantly Chinese children
HAL/Essilor Stellest spectacle lenses	Highly aspherical lenslets impose myopic defocus while correcting central refractive error	71% reduction in SER progression and 53% reduction in axial elongation	Spectacle-lens adaptation issues; non-invasive	Unknown/likely low relative to pharmacologic cessation, but long-term data needed	FDA De Novo DEN250016 granted Sept. 25, 2025; first U.S. eyeglass lens indicated to slow pediatric myopia progression	**Moderate** — regulatory-reviewed clinical data; post-market experience still developing
Low-dose atropine (0.01%–0.05%)	Muscarinic antagonist effects on retinal/scleral pathways; concentration-dependent efficacy	LAMP 2-year SER progression: 0.55 D, 0.85 D, 1.12 D for 0.05%, 0.025%, 0.01%	Photophobia and near blur increase with concentration; allergy uncommon	Significant, especially with higher concentrations; 0.01% showed lower rebound in ATOM2	Myopia-control use remains off-label in several jurisdictions, depending on product and country	**High** — large RCTs and longer follow-up, but efficacy varies by concentration and population
Multifocal soft contact lenses / MiSight	Peripheral or simultaneous myopic defocus through concentric or dual-focus optical zones	59% less SER progression and 52% less axial elongation	Contact-lens discomfort, dryness, rare microbial keratitis	Moderate; progression may accelerate after discontinuation	MiSight FDA PMA approved in 2019 for specified children aged 8–12 years at initiation	**Moderate–High** — 3-year RCT evidence and regulatory authorization
Outdoor light exposure	High illuminance, broad spectrum, reduced near-work intensity, longer viewing distance, circadian and dopamine-related mechanisms	Meta-analyses show reduced incident myopia; effect on established myopia progression is smaller and less certain	Safest intervention overall; UV protection and heat precautions advised	None observed	Not a device; encouraged in multiple guidance/public-health contexts rather than “globally endorsed” by a single authority	**Moderate** — large cohorts and school RCTs; confounding remains important

AE, adverse event; GRADE, evidence quality grading; NNT, number needed to treat; IDE, Investigational Device Exemption. Evidence graded: High, multiple consistent RCTs with long follow-up and diverse populations; Moderate, RCTs with limitations; Low, observational data only. Bold values indicate the overall GRADE evidence-quality rating for each intervention.

### Violet light–transmitting eyewear

Violet light has attracted interest because ordinary modern spectacle and window materials may block wavelengths in the 360–400 nm range, and animal studies implicate OPN5 in violet-light-dependent suppression of lens-induced myopia (38, 42). The key human RCT by Mori et al. evaluated violet-light-transmitting eyeglasses, not contact lenses, in children aged 6–12 years over 2 years (59). The trial reported 21.4% suppression of axial elongation over 2 years in the violet-light-transmitting eyeglass group, but the overall effect was not statistically significant without subgrouping; significant differences were reported in specified subgroups such as children with shorter near-work time and children who had not previously worn eyeglasses (59). Thus, violet-light eyewear remains biologically plausible but clinically less established than RLRL or validated defocus-based optical interventions. Claims of large one-year effects, contact-lens delivery, or broad efficacy across all children should be avoided unless supported by new trials.

### Optical defocus lenses: DIMS and multifocal designs

Defocus-incorporated multiple segments (DIMS) spectacle lenses employ concentric annular zones imparting +3.50 D peripheral myopic defocus while maintaining central correction. A 2-year RCT (N, 160) in Hong Kong schoolchildren (8–13 years) demonstrated a 52% reduction in myopia progression (−0.41 ± 0.04 vs. −0.85 ± 0.05 D; p < 0.001) and 62% reduction in axial elongation (0.21 ± 0.03 vs. 0.55 ± 0.04 mm; p < 0.001) (60). Ortho-k lenses produce similar outcomes (40–50% axial control) with overnight corneal reshaping but require stringent hygiene to avoid adverse events (61).

### Pharmacological agents: low-dose atropine

Low-dose atropine eye drops remain among the most widely studied pharmacological interventions for pediatric myopia control. In the 2-year LAMP study, mean spherical equivalent progression was 0.55 ± 0.86 D with 0.05% atropine, 0.85 ± 0.73 D with 0.025% atropine, and 1.12 ± 0.85 D with 0.01% atropine; corresponding axial length changes were 0.39 ± 0.35 mm, 0.50 ± 0.33 mm, and 0.59 ± 0.38 mm, respectively (62). These data supported a concentration-dependent effect, with 0.05% atropine showing approximately double the efficacy of 0.01% atropine over 2 years (62). ATOM2 remains important for longer-term atropine interpretation and rebound after cessation (63). Five-year follow-up revealed a rebound effect after cessation, with 0.01% atropine exhibiting the lowest rebound compared to higher concentrations (63).

### Combination therapies

Recent RCTs explore synergistic combinations: RLRL + orthokeratology demonstrated 0.06 mm axial growth versus 0.23 mm with ortho-k alone at 12 months (p < 0.01) (64). Trials investigating atropine + multifocal soft contact lenses show 70% progression reduction versus 50% with monotherapy (65). Protocol optimization continues, with AI-driven models guiding personalized regimens based on baseline refractive error, outdoor activity levels, and genetic markers.

## Safety profiles, regulatory considerations, and risk assessment

The rapid clinical adoption of spectral light therapies, particularly RLRL, has necessitated comprehensive safety evaluation across multiple domains: photometric exposure limits, clinical adverse events, regulatory oversight, and long-term ocular health implications. This section provides an analysis of safety data from clinical trials, laboratory studies, and post-market surveillance.

### Photometric safety analysis and maximum permissible exposure

Detailed photometric characterization of commercial RLRL devices reveals concerning findings regarding retinal exposure limits. The American National Standards Institute (ANSI Z136.1-2014) establishes maximum permissible exposure (MPE) limits based on wavelength, exposure duration, and retinal spot size to prevent thermal and photochemical retinal damage. Two widely used devices—the Sky-n1201a and Future Vision—demonstrate fundamentally different optical designs with distinct safety profiles.

The Sky-n1201a delivers coherent 654 nm laser light as a point source, producing corneal irradiance of 1.17 mW/cm² at 10 cm viewing distance through a 7 mm aperture (66). With a 2 mm pupil, retinal irradiance reaches 7.2 W/cm², approaching the photochemical damage threshold within 0.55–7.0 seconds depending on pupil size. For pupils ≥4.25 mm, thermal damage MPE is exceeded within 0.41–10 seconds, well below the standard 3-minute treatment protocol (66). These calculations assume direct retinal viewing which is the intended clinical application.

The Future Vision device employs an extended LED source subtending 0.75° × 0.325° visual angle, reducing peak irradiance through spatial distribution. With 652 nm wavelength and 0.06 mW total power, corneal irradiance is 0.624 mW/cm² and retinal irradiance 0.08 W/cm² (2 mm pupil). Photochemical damage MPE ranges from 50–625 seconds across pupil sizes, suggesting greater safety margins but still approaching limits during 3-minute exposures (66).

### Clinical safety outcomes from systematic review evidence

A comprehensive systematic review encompassing 20 studies and 2, 380 participants (median age 10.2 years, treatment duration 6–24 months) provides the most robust clinical safety data available (67). The pooled adverse event incidence was 0.088 per 100 patient-years (95% CI: 0.02–0.50), significantly lower than orthokeratology (20.6/100 patient-years) and low-dose atropine (7.32/100 patient-years) (67).

### Transient visual effects, structural retinal changes and serious adverse events

The most common adverse event is temporary afterimages following treatment, reported in 15–45% of subjects across studies. Afterimage duration ranges from 30 seconds to 10 minutes (median: 3.2 minutes), with longer durations correlating with higher device power settings (68). Importantly, afterimage persistence >15 minutes occurred in <0.5% of treatments and resolved spontaneously without intervention.

High-resolution adaptive optics imaging has introduced new safety considerations. In a multicenter cohort study of 99 children with myopia, RLRL users showed lower cone density within 0.5 mm of the foveal center, most notably in the temporal region (58). At 0.3 mm temporal eccentricity, the mean cone-density difference compared with controls was -2.1 × 10³ cells/mm² (95% CI, -3.68 to -0.59 × 10³ cells/mm²; p = 0.003) (58). In addition, 11 eyes exhibited abnormal low-frequency, high-brightness signals near the fovea, and 1 eye had a small OCT cystoid abnormality in the ganglion cell layer that resolved 3 months after discontinuing RLRL therapy (58). These findings do not establish incidence in all users or prove irreversible harm, but they reinforce the need for longer-term safety studies and careful monitoring.

Cases have been reported with patients experiencing bilateral visual acuity decline with optical coherence tomography abnormalities including hyperreflective foci and outer retinal disruption after 18 months of RLRL therapy (69). Complete recovery occurred after treatment discontinuation, suggesting reversible photoreceptor dysfunction rather than permanent structural damage.

## Comparative risk–benefit profile of myopia control interventions

Quantitative risk–benefit modeling using number-needed-to-treat (NNT) and number-needed-to-harm (NNH) provides a standardized framework for comparing myopia control modalities. When modeled for prevention of 1.0 diopter (D) of myopic progression over 12 months, repeated low-level red-light (RLRL) therapy demonstrates a favorable therapeutic index relative to established interventions (70, 71). **Table 2** compares RLRL therapy, orthokeratology, DIMS spectacle lenses, and low-dose atropine across: efficacy (axial length control %), safety profile, proposed mechanism, rebound effects, regulatory approval status, and GRADE evidence quality.

RLRL therapy exhibited the lowest NNT, indicating higher treatment efficacy, while maintaining comparatively high NNH values for both transient (afterimages) and structural adverse outcomes. In contrast, orthokeratology and low-dose atropine demonstrated higher NNTs and lower NNHs for commonly reported adverse effects, reflecting narrower safety margins (**Table 3**). The modeled risk–benefit ratio favors RLRL therapy by approximately 12.7–21.4-fold in children with moderate-to-high myopia (−3.0 to −8.0 D), although this advantage diminishes in low myopia due to reduced absolute treatment benefit (70).

**Table 3:** Quantitative risk–benefit comparison of myopia control therapies.

Intervention	NNT (1 D Control / 12 mo)	Primary Adverse Event	NNH (Primary)	Serious Adverse Event	NNH (Serious)
RLRL therapy	2.3	Afterimages	50–115	Structural retinal changes	556
Orthokeratology	4.1	Corneal staining	12	Infectious keratitis	2,500
0.01% Atropine	5.8	Photophobia	3.2	Allergic reactions	25

NNT/NNH estimates can vary by baseline risk, age, myopia severity, adherence, comparator, and surveillance intensity.

## Pediatric safety and developmental considerations

Children represent a uniquely vulnerable population due to ongoing ocular development, prolonged cumulative exposure potential, and behavioral factors that may influence treatment delivery. Key pediatric-specific safety considerations and their clinical implications are summarized in **Table 3**. Smaller pupil diameters in children (typically 2–5 mm) may reduce peak retinal irradiance; however, photochemical risk is governed primarily by cumulative light dose rather than instantaneous irradiance, limiting the protective effect of pupil size (72). In addition, increased crystalline lens transparency in pediatric eyes may result in greater retinal light transmission than predicted by adult-based safety models, potentially underestimating true retinal exposure (**Table 4**). Behavioral compliance represents an additional challenge in pediatric populations. Difficulty maintaining precise fixation during treatment may lead to uneven retinal exposure patterns (73). While involuntary eye movements may reduce localized peak exposure, they may also increase the total retinal area exposed, thereby altering cumulative dose distribution. Importantly, the absence of longitudinal data extending beyond five years limits conclusions regarding delayed retinal effects or interactions between cumulative exposure and long-term retinal aging.

**Table 4:** Pediatric safety considerations and monitoring implications.

Factors	Key consideration	Clinical implication
Ocular development	Smaller pupils reduce peak irradiance but not cumulative dose	Photochemical risk remains
Lens transparency	Higher retinal transmission in children	Adult safety models may underestimate exposure
Behavioral compliance	Fixation instability and eye movement	Uneven retinal dose distribution
Long-term exposure	No data beyond 5 years	Unknown delayed or cumulative effects

### Regulatory affairs and international standards

Regulatory approaches to RLRL devices vary substantially across jurisdictions, reflecting differing risk tolerance thresholds for pediatric light-based therapies. China’s National Medical Products Administration has recently reclassified RLRL devices from Class II to Class III medical devices, mandating enhanced post-market surveillance following emerging safety concerns (74). In the European Union, CE marking under the Medical Device Regulation requires comprehensive clinical evaluation and risk management documentation, with additional scrutiny for pediatric indications. In the United States, no RLRL device has received FDA approval for myopia control, and all clinical use remains investigational under Investigational Device Exemption requirements. Australia and New Zealand have adopted a conditional approval framework, incorporating mandatory post-market surveillance, prescriber certification, and periodic safety reporting.

## Recommended safety monitoring and risk mitigation strategies

Based on available safety data and current regulatory guidance, consensus recommendations emphasize structured clinical monitoring across the treatment lifecycle, combined with device-level engineering controls to mitigate exposure-related risks. A phased monitoring framework encompassing pre-treatment assessment, active treatment surveillance, and post-treatment follow-up is recommended to ensure early detection of adverse effects and to limit cumulative retinal exposure (75). These clinical safeguards are complemented by emerging device design enhancements aimed at improving dose precision and treatment safety (**Table 5**).

**Table 5:** Recommended clinical monitoring and engineering-based safety measures for RLRL therapy.

Phase/domain	Safety measure	Purpose/rationale
Pre-treatment assessment	Comprehensive ophthalmic examination with fundus photography and spectral-domain OCT	Establish baseline retinal structure and exclude pre-existing pathology
	Baseline cone density assessment using adaptive optics (where available)	Detect subtle photoreceptor changes and enable longitudinal comparison
	Patient and guardian education on warning symptoms	Promote early reporting of adverse visual phenomena
During treatment	Monthly clinical assessments for first 3 months, followed by quarterly reviews	Enable early detection of intolerance and dose-related effects
	Home symptom diary documenting afterimage duration and visual disturbances	Capture transient symptoms not observed during clinic visits
	Immediate treatment cessation if afterimages persist >15 minutes or visual acuity declines	Prevent cumulative retinal injury from repeated exposure
Post-treatment follow-up	Continued monitoring for 6 months after treatment cessation	Identify rebound myopia progression or delayed adverse effects
	Annual retinal examination for patients with >12 months cumulative exposure	Monitor for long-term or cumulative retinal changes
Device engineering controls	Real-time infrared pupillometry with dynamic power adjustment	Maintain consistent retinal irradiance despite pupil size variability
	Automated fail-safe shutdown triggered by fixation loss, blinking, or excessive eye movement	Prevent unintended or uncontrolled retinal exposure
	Personalized dosimetry incorporating fundus reflectance, lens transmission, and retinal pigmentation	Optimize therapeutic dose while preserving safety margins

Pre-treatment evaluation should include a comprehensive ophthalmic examination with multimodal retinal imaging to establish structural and functional baselines. During active treatment, intensified monitoring in the initial treatment phase is warranted to identify early intolerance or abnormal visual responses. Extended post-treatment surveillance is advised, particularly for patients with prolonged cumulative exposure, to detect delayed or rebound effects. In parallel, incorporation of real-time pupillary monitoring, automated fail-safe mechanisms, and personalized dosimetry represents a critical engineering pathway to reducing inter-individual variability in retinal light exposure (**Table 4**).

## Environmental epidemiology and public health implementation

Population-level myopia prevention depends on scalable, low-cost environmental interventions that complement individual-based therapies. Over the past two decades, epidemiological evidence has consistently identified outdoor light exposure as a major protective factor against myopia onset (4, 5, 76–79). Large cohort and interventional studies demonstrate robust inverse associations between time spent outdoors and myopia incidence across diverse populations (5). For example, longitudinal data indicate that each additional hour of weekly outdoor exposure is associated with a measurable reduction in myopia risk after adjustment for near work, socioeconomic status, and parental myopia (5). Randomized school-based interventions further support causality, with increased outdoor recess time producing clinically meaningful reductions in incident myopia. Objective light dosimetry studies using wearable sensors reinforce these findings, identifying cumulative daily light exposure thresholds associated with substantially lower myopia risk. Collectively, these data have informed national public health guidelines in multiple regions, recommending a minimum of two hours of outdoor activity per day for school-aged children. Interpretation of these epidemiological associations must, however, account for significant confounding. Physical activity coinciding with outdoor time may independently influence axial growth through postural and accommodative mechanisms unrelated to light per se. Socioeconomic status affects access to outdoor environments and is independently associated with educational pressure—strongly linked to myopia in East Asian populations. Regional lifestyle differences, including dietary patterns, screen time norms, school hours, and population density, vary substantially across studies and complicate cross-cultural generalization. Future cohort studies employing wearable light dosimetry with accelerometry and ecological momentary assessment will be essential for disentangling the contributions of luminous intensity, spectral composition, and physical activity to myopia risk reduction.

When outdoor access is constrained, indoor environmental modification represents a viable strategy. Typical indoor illuminance levels (100–500 lux) fall well below those associated with myopia protection, whereas classroom retrofitting with high-intensity lighting (1, 500–3, 000 lux) has been shown to significantly reduce axial elongation and slow myopia progression in controlled trials (80). Beyond illuminance, spectral composition appears biologically relevant, with short-wavelength–enriched lighting linked to biomarkers consistent with dopamine-mediated retinal signaling (44). At the community level, urban planning initiatives that prioritize access to green spaces and structured outdoor activity programs have demonstrated modest but consistent reductions in myopia progression (81). Economic evaluations further indicate that outdoor activity promotion and indoor lighting interventions are highly cost-effective relative to pharmacologic approaches, particularly in resource-limited settings. Together, these findings support environmental and architectural strategies as foundational components of population-level myopia control programs (82).

## Future directions and technological innovation

Advancement of light-based myopia control will depend on the integration of precision-engineered delivery systems, individualized dosimetry, and data-driven treatment optimization. Next-generation RLRL platforms are expected to incorporate real-time pupillometry and eye-tracking to dynamically modulate retinal irradiance in response to pupil diameter and gaze stability, thereby reducing inter- and intra-subject variability in delivered dose. The use of narrow-band micro-LED arrays with individually addressable pixels may enable spatially distributed retinal stimulation, mitigating localized photochemical risk while preserving therapeutic efficacy. Closed-loop control systems incorporating ocular surface temperature monitoring and retinal reflectance feedback could further ensure compliance with ANSI-specified maximum permissible exposure limits at both per-session and cumulative dose levels. In parallel, adaptive optics–enabled correction of individual ocular wavefront aberrations, combined with spectral tuning using multi-wavelength light sources, may facilitate personalized chromatic stimulation protocols tailored to longitudinal chromatic aberration profiles, cone distributions, and retinal transmission characteristics.

Digital and computational innovations are likely to extend light-based interventions beyond dedicated devices into continuous, behavior-integrated platforms. Augmented reality–based chromatic modulation delivered via smart glasses could provide real-time peripheral defocus and spectral contrast manipulation during routine visual tasks, while compensating for reduced outdoor light exposure in indoor environments. Machine learning–driven predictive models trained on multimodal clinical data—including genetic predisposition, baseline refractive status, lifestyle factors, and early treatment response—may enable stratification of myopia progression risk and dynamic optimization of treatment schedules. Reinforcement learning frameworks incorporating longitudinal axial length or refractive feedback could further adapt exposure parameters over time. These technological advances will facilitate rational combination therapies integrating RLRL with optical defocus strategies, pharmacologic agents, and behavioral interventions. Future clinical trials employing adaptive, factorial designs will be essential to identify synergistic dosing hierarchies, maximize long-term efficacy, and define safety boundaries across diverse pediatric populations. The AI-driven and closed-loop therapeutic strategies described in this section remain speculative and prospective in nature. None have been validated in large-scale clinical trials; they represent promising research directions whose safety and efficacy profiles remain to be determined.

## Conclusions

The convergence of optical physics, retinal neurobiology, and digital health has fundamentally expanded the therapeutic landscape for myopia control, enabling biologically informed interventions that extend beyond conventional optical or pharmacologic correction. Evidence synthesized in this review indicates that light-based strategies—particularly repeated low-level red-light therapy—demonstrate clinically meaningful efficacy in slowing myopia progression, supported by emerging mechanistic insights into retinal signaling pathways and ocular growth regulation. Nonetheless, the long-term success of such interventions will depend on rigorous attention to safety, dose optimization, and inter-individual variability, particularly in pediatric populations with prolonged cumulative exposure.

Future progress will require coordinated, interdisciplinary efforts integrating advances in photonics, adaptive device engineering, computational modeling, and population health implementation. Translation from controlled clinical efficacy to real-world effectiveness will necessitate standardized monitoring frameworks, personalized treatment algorithms, and equitable deployment strategies informed by health economic evaluation. Ultimately, addressing the global myopia epidemic demands a shift from reactive refractive correction toward proactive, preventive paradigms grounded in environmental modification and precision therapeutics. Light-based interventions represent a compelling pathway within this framework, with the potential to preserve visual function and quality of life across the lifespan if implemented with scientific rigor, regulatory oversight, and global public health alignment.
